# Mesenchymal stem cells increase expression of heme oxygenase-1 leading to anti-inflammatory activity in treatment of acute liver failure

**DOI:** 10.1186/s13287-017-0524-3

**Published:** 2017-03-20

**Authors:** Zhi-heng Zhang, Wei Zhu, Hao-zhen Ren, Xin Zhao, Shuai Wang, Hu-cheng Ma, Xiao-lei Shi

**Affiliations:** 1 Department of Hepatobiliary Surgery, The Affiliated Drum Tower Hospital of Nanjing University Medical School, Nanjing, China; 2 Department of Anesthesiology, The Affiliated Drum Tower Hospital of Nanjing University Medical School, Nanjing, China; 3 Department of Hepatobiliary Surgery, Drum Tower Clinical Medical College of Nanjing Medical University, Nanjing, China

**Keywords:** Mesenchymal stem cells, Heme oxygenase-1, Acute liver injury, PMNs, Neutrophils, Inflammation, NF-κB

## Abstract

**Background:**

Mesenchymal stem cells (MSCs) have been studied for the treatment of acute liver failure (ALF) for several years. MSCs may exert their effect via complex paracrine mechanisms. Heme oxygenase (HO) 1, a rate-limiting enzyme in heme metabolism, exerts a wide range of anti-inflammatory, anti-apoptotic and immunoregulatory effects in a variety of diseases. However, the relationship between MSCs and HO-1 in the treatment of ALF is still unclear. We investigated the preventive and therapeutic potential of intravenously administered BMSCs.

**Methods:**

Bone marrow-derived mesenchymal stem cells (BMSCs) obtained from Sprague–Dawley rats were isolated and cultured. We employed BMSCs, hemin (a HO-1 inducer) and zinc protoporphyrin (ZnPP, the HO-1 activity inhibitor) in D-galactosamine (D-Gal)/lipopolysaccharides (LPS)-induced ALF rats. Rats were sacrificed at days 1, 3, 5, and 7 post-transfusion, respectively. Blood samples and liver tissues were collected. Hepatic injury, HO-1 activity, chemokines, inflammatory cytokines, the number and oxidative activity of neutrophils, ki67, and TUNEL-positive cells were evaluated.

**Results:**

HO-1 induction or BMSCs transplantation attenuated D-galactosamine/lipopolysaccharide-induced increases in alanine aminotransferase, aspartate aminotransferase, total bilirubin (TBIL), ammonia, and inflammatory cytokines. Treatment with hemin or BMSCs also inhibited neutrophil infiltration, oxidative activity, and hepatocyte apoptosis. The protective effect of BMSCs was partially neutralized by ZnPP, suggesting the key role of HO-1 in the process.

**Conclusions:**

These findings may correlate with inhibition of nuclear factor-κ B activation. BMSCs ameliorated ALF by increasing the HO-1 expression, which reduced PMN infiltration and function, and played an important anti-inflammatory and anti-apoptotic role.

**Graphical Abstract:**

Proposed mechanism by which BMSCs reduce inflammation, neutrophil activation, and hepatocyte apoptosis and promote hepatocyte proliferation via HO-1. BMSCs increase HO-1 expression in liver via Nrf2. HO-1 protects against LPS/D-Gal-induced ALF by inhibiting neutrophil infiltration and inflammatory burst, and hepatocyte apoptosis and necrosis. HO-1 also promotes hepatocyte proliferation.
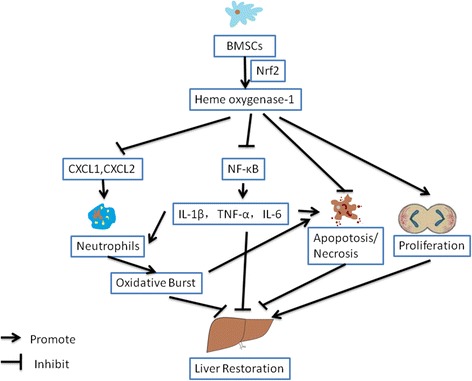

**Electronic supplementary material:**

The online version of this article (doi:10.1186/s13287-017-0524-3) contains supplementary material, which is available to authorized users.

## Background

Acute liver failure (ALF) is a clinical manifestation of sudden and severe hepatic injury associated with a high mortality rate. Orthotopic liver transplantation (OLT) is the only effective treatment [1, 2]. However, the shortage of available donor livers and multiple postoperative complications limit widespread clinical application of OLT [3]. No artificial liver device has emerged as a way to bridge patients with acute liver disease to transplantation or recovery. Recently, some stem cell therapies have shown promise in treating acute liver failure and drug-induced liver injury [4–6].

Polymorphonuclear neutrophils (PMNs) are part of the innate immune system and migrate to sites of inflammation. In immune and inflammatory disorders, PMNs are released from the marrow and circulate to liver. Inflammatory mediators, such as tumor necrosis factor (TNF)-α, interleukin (IL)-1β and IL-8 released from dying or dead hepatocytes, are potent promoters of PMN infiltration into the liver parenchyma [7, 8]. These effects trigger PMN activation, including prolonged adherence-dependent oxidative stress and degranulation [9]. Neutrophils express Fas ligand, and they play a key role in killing microorganisms or damaged cells, thereby limiting disease spread. These same functions mean that neutrophils can cause hepatocytes apoptosis [10]. PMNs are essential for a healthy liver although excessive activation of neutrophils perpetuates liver injury.

Mesenchymal stem cells (MSCs) are stroma-derived cells occurring in several tissues, such as bone marrow, adipose tissue, skeletal muscle, synovium, umbilical cord, and cord blood [11]. The ability of MSCs to modulate cell function in innate and adaptive immune systems suggests a therapeutic role in inflammatory diseases [12]. In addition to promoting differentiation in regeneration therapy, MSCs show low immunogenicity. They play an immunoregulatory role in the secretion of mediators, including transforming growth factor (TGF)-β, indoleamine 2,3-dioxygenase, inducible nitric oxide synthase (iNOS), prostaglandin E2 (PGE2), IL-10, and TNF-α-stimulating gene (TSG)-6 [13]. These properties suggest great therapeutic potential for the treatment of immune and inflammatory disorders. However, the underlying mechanisms of MSCs in ALF remain elusive.

Heme oxygenase (HO) comprises a group of ubiquitous enzymes [14]. HO is expressed in three isoforms: HO-1, 2, and 3. HO-1 is a rate-limiting enzyme in heme metabolism, catalyzes heme into CO, free iron, and biliverdin, and exerts a wide range of anti-inflammatory, anti-apoptotic, and immunoregulatory effects in a variety of diseases [14–16]. It is upregulated by pharmacological agents in D-galactosamine (D-Gal)/lipopolysaccharide (LPS)-induced liver injury [17]. A recent study suggested that MSCs transplantation upregulates the expression of HO-1 [13].

Several studies have demonstrated the therapeutic effect of MSCs in ALF [4–6]. However, the relationship between HO-1, neutrophils, and the therapeutic effect of MSCs in ALF has not been demonstrated. Therefore, this study sought to determine whether MSCs protected against ALF by inducing HO-1 expression in liver and suppressing PMN activation.

## Methods

### Animals

Male Sprague–Dawley rats aged 3–4 weeks and weighing 90–100 g each were used as BMSC donors. Male rats aged 6–7 weeks and (weighing 190–200 g each were used as BMSC recipients. All the rats were purchased from the Laboratory Animal Center of the Affiliated Drum Tower Hospital of Nanjing University Medical School, China, housed in a ventilated cabinet under controlled air pressure and temperature conditions, and exposed to alternating 12 h light/dark cycles. The rats were provided with sterile water and standard pellets of rodent diet. The animals were sacrificed.

### Preparation of bone marrow-derived mesenchymal stem cells (BMSCs)

BMSCs obtained from Sprague–Dawley rats were isolated and cultured according to an established protocol [5]. The rats were sacrificed by cervical dislocation, the femurs and tibias were excised and the soft connective tissue was removed. The bone marrow cells were harvested by flushing the marrow cavity with complete culture medium. Cells were collected by gradient centrifugation over a Ficoll Histopaque layer (20 min, 400 × *g*, density 1.077 g/ml) and seeded at a density of 1 × 10^6^ cells/cm^2^ in growth medium containing low-glucose Dulbecco's modified Eagle's medium (DMEM; Gibco, Grand Island, NY, USA) supplemented with 10% fetal bovine serum, penicillin (100 U/ml), and streptomycin (100 mg/ml). The nonadherent cells were removed after the first 24 h and changed every 3–4 days thereafter. When the cells reached 80% confluence, they were detached using 0.25% (w/v) trypsin-ethylenediaminetetraacetic acid and replated at a density of 1 × 10^4^cells/cm2 for expansion. We used flow cytometry (FACS Aria II; BD Biosciences, San Jose, CA, USA) to identify the MSCs. We used antibodies targeting rat antigens cluster of differentiation (CD)29, CD34, CD44, CD45, and CD90 (BD Biosciences). Positive cells were counted and compared with the signal corresponding to the immunoglobulin isotype. The BMSCs obtained between the third and seventh passages were used.

### Experimental design and animal groups

ALF was induced by intraperitoneal injection of 0.8 g/kg D-galactosamine (D-Gal) and 20 μg/kg LPS. The rats were randomly divided into five groups: (1) control; (2) ALF; (3) ALF + MSC; (4) ALF + MSC+ zinc protoporphyrin (ZnPP); and (5) ALF + hemin. The control rats were injected with an equal volume of normal saline in parallel. One hour after ALF induction, either phosphate-buffered saline (PBS) or 10^6^ MSCs in a volume of 1.0 mL were transfused into the caudal tail vein over a period of 3 min. Hemin (40 μmol/ kg body weight; Sigma–Aldrich, St. Louis, MO, USA) or ZnPP (50 μmol/kg body weight; Sigma–Aldrich) were administered intraperitoneally 1 h after induction of liver injury. The doses of D-Gal, LPS, hemin, and ZnPP were based on our preliminary studies. Rats were evaluated every 6 h and euthanized if they appeared moribund.

### Survival study

Ten rats in each group were used for the survival study. Rats that lived for more than 12 days after transplantation were considered as survivors.

### Collection of serum samples and hepatic tissue specimens

Rats were euthanized on days 1, 2, 3, 5, and 7 post-transfusion. To detect serum cytokines on days 0, 1, 2, 4, and 6 after infusion of BMSCs, blood samples were collected from the tail vein 24 h before euthanasia. Liver tissues were excised and processed for further RNA analysis and Western blot. The remaining tissue was fixed and processed for histology and immunohistochemistry.

### Measurement of hepatic enzyme and cytokine levels after cell transfusion

The plasma levels of alanine aminotransferase (ALT) and aspartate aminotransferase (AST) after treatment were measured with an automated biochemical analyzer (iMagic-M7; Mindray, Shenzhen, China). The levels of plasma IL-1β, IL-6, and TNF-α were detected using a commercially available ELISA kit (eBioscience, San Diego, CA, USA).

### Measurement of mRNA expression in hepatic tissues by quantitative reverse transcriptase polymerase chain reaction (RT-PCR)

Total RNA was isolated from frozen liver using TRIzol reagent (Invitrogen, Carlsbad, CA, USA). First-strand cDNA was synthesized using the Superscript II Reverse Transcriptase Kit (Invitrogen). Quantitative (q) PCR was performed using Power SYBR Green PCR Master Mix (Takara, Tokyo, Japan). The relative level of gene expression was normalized to that of an internal control (β-actin) and calculated using the 2^−ΔΔCT^ method. The primer sequences were as follows:

HO-1 sense primer sequence: 5'-ACCCCACCAAGTTCAAACAG-3'; HO-1 antisense prime sequence: 5'-GAGCAGGAAGGCGGTCTTAG-3'; IL-1β sense primer sequence: 5'-TCAATCAGCCCTTTACTGAAGATG-3'; IL-1β antisense prime sequence: 5'-TGCTTGACGATCCTTATCAATTTG-3'; IL-6 sense primer sequence: 5'-CAAAGCCAGAGTCATTCAAGC-3'; IL-6 antisense prime sequence: 5'-GGTCCTTAGCCACTCCTTCTGT-3'; TNF-α sense primer sequence: 5'-CCCAATCTGTGTCCTTCTAACT-3'; TNF-α antisense prime sequence: 5- CACTACTTCAGCGTCTCGTGT-3'; CXCL-1 sense primer sequence: 5-TCTTTCTGGCTTAGAACAAAGGGGC-3; CXCL-1 antisense prime sequence: 5-AGTAAAGGTAGCCCTTGTTTCCCCC-3; CXCL-2 sense primer sequence: 5-TCATAGCCACTCTCAAGGG -3; CXCL-2 antisense prime sequence: 5-TTGGTTCTTCCGTTGAGGG-5; CXCL12 sense primer sequence: 5′-GAT TGT AGC CCG GCT GAA GA -3′; CXCL12 antisense prime sequence: 5′-TTC GGG TCA ATG CAC ACT TGT -3; β-actin sense primer sequence: 5'-GCGCTCGTCGTCGACAACGG-3'; β-actin antisense primer sequence: 5'-GTGTGGTGCCAAATCTTCTCC-3'.

Experiments were performed in replicate using three different samples of each group, and technical triplicates were obtained for the analysis of each gene expression. The mean relative expression of each gene in the groups was used for statistical analysis.

### Histological and immunohistochemical study

At the end of the study, livers were removed and weighed. The liver samples were fixed in 4% paraformaldehyde for 24 h before processing for histological and immunohistochemical analyses. Fixed liver samples were dehydrated and paraffin-embedded. Three fragments, each measuring less than 3 mm in thickness, were obtained from each liver. Sections were stained with hematoxylin and eosin for pathological assessment. Apoptosis was assessed by TdT-mediated dUTP nick end-labeling (TUNEL) staining, using a Cell Death Detection Kit (Roche, Mannheim, Germany). Cells testing positive for proliferating cell nuclear antigen (Ki67) were detected using specific antibodies (Aviva Systems Biology, Beijing, China). Formalin-fixed paraffin-embedded liver sections were deparaffinized and stained for HO-1 and myeloperoxidase (MPO). Images were captured, and the labeled cell area manually quantified by two independent operators using Image-Pro Plus software (Media Cybernetics, Bethesda, MD, USA), five different fields were taken in each individual sample. Then the average number of the positive cells was calculated.

### Histological analysis

The histological changes were evaluated according to the method reported by Camargo et al. [18]. The stained sections were graded as follows: grade 0, minimal or no evidence of injury; grade 1, mild injury with cytoplasmic vacuolation and focal nuclear pyknosis; grade 2, moderate to severe injury with extensive nuclear pyknosis, cytoplasmic hypereosinophilia, and loss of intercellular borders; grade 3, severe necrosis with disintegration of hepatic cords, hemorrhage, and neutrophil infiltration.

### Western blotting analysis of Bcl-2, Bax, Nrf2, and HO-1 expression in liver tissue

The total, cytosolic, and nuclear protein content of frozen hepatic samples was extracted according to the method described in the protein extraction kit (Active Motif, Carlsbad, CA, USA). Protein concentrations were determined using BCA protein assay kit (Sigma–Aldrich). Protein extracts were fractionated on 12% SDS-PAGE and transferred to a nitrocellulose membrane. The membrane was blocked with 5% (w/v) fat-free milk in Tris-buffered saline (TBS) containing 0.05% Tween 20, followed by incubation with a rabbit anti-HO-1 polyclonal antibody (1:2000), rabbit anti-Bcl-2 polyclonal antibody (1:1000) or rabbit anti-Bax polyclonal antibody (1:1000) at 4 °C overnight. The membrane was treated with horseradish peroxidase-conjugated goat anti-rabbit secondary antibody (1:10,000). Antibody binding was visualized with an ECL chemiluminescence system and short exposure of the membrane to X-ray films (Kodak, Tokyo, Japan). Signal intensities were quantified by densitometry using Image J software (NIH, Bethesda, MD, USA).

### Measurement of nuclear factor (NF)-κB (p65) binding ability

The nuclear protein fraction in each experimental animal was used for the NF-κB-DNA binding assay. The NF-κB-DNA binding activity was assessed using an NF-κB (p65) transcriptional factor ELISA kit (Cayman Chemical Company, Ann Arbor, MI, USA).

### Measurement of HO-1 activity

HO-1 activity was determined via bilirubin formation as described by Hu et al. [19]. The reaction mixture consisted of 200 μL 4 mmol/L liver supernatant, 50 μL 1 mmol/L liver cytosol, 20 μL 1 mmol/L heme B solution, 200 μL 2.75 mmol/L β-NADPH solution, and 530 μL 2 mmol/L MgCl_2_ in 100 mmol/L phosphate buffer (pH 7.4). The samples were incubated in a 37 °C water bath in the dark for 1 h. The reaction was stopped by placing the samples in ice. An NADPH-free reaction mixture was used as a control. The absorbance of the samples was measured using a UV/visible spectrophotometer (Ultrospec 2000; Thermo Fisher Scientific, Waltham, MA, USA) at 464 nm and 530 nm. Bilirubin levels were calculated from the difference in absorbance at 464 nm and 530 nm. The values were expressed as pmol bilirubin/mg protein formed/h.

### Malondialdehyde (MDA) and MPO activity

Lipid peroxidation in the livers was determined by measuring the thiobarbituric acid-reactive substances in the liver tissue homogenate using an MDA assay kit (Beyotime Institute of Biotechnology, Beijing, China). Liver MPO activity was determined using an MPO activity assay kit (Jiancheng Biochemistry Co., Nanjing, China). Three liver samples in each pup at different time points were used for each analysis.

### Oxidative activity

Oxidative activity was determined by staining with dihydrorhodamine 123 (DHR123; Sigma–Aldrich), a nonfluorescent agent converted by cellular oxidation to the fluorescent dye rhodamine 123 (R123). Heparinized whole blood samples (100 μL) from each group were incubated with DHR123 (1.0 μM) in 400 μL RPMI 1640 at 37 °C under sterile conditions. Leukocytes were isolated by ammonium chloride lysis of red blood cells. Cells in these gates were analyzed for fluorescence intensity. Cell-associated R123 fluorescence within the three populations was determined using a flow cytometer (FACS Aria II; BD Biosciences). Background fluorescence in samples incubated without R123 was subtracted from the total fluorescence to determine the oxidative activity in each individual sample.

### Statistical analysis

All the data were expressed as means ± standard deviation (SD) and compared via analysis of variance followed by a Student’s *t* test. In the mortality study, time-to-survival data were analyzed using the Kaplan–Meier method and compared via the log-rank test. Differences between values were considered significant at *p* < 0.05.

## Results

### BMSC isolation and characterization

BMSCs were isolated from Sprague–Dawley rats. The adherent cells showed a colony-like distribution after inoculation into the culture flask, as seen under a phase-contrast microscope. These cells exhibited a spindle-shaped, fibroblastic morphology (Additional file 1: Figure S1A). The BMSCs were identified by flow cytometry. Cells were incubated with fluorescein isothiocyanate (FITC)-conjugated antibodies. The BMSCs isolated from rat were positive for CD29 (98.9%), CD44 (97.1%), and CD90 (99.2%), and negative for CD34 (0.8%) and CD45 (2.0%), which suggested high purity after the third passage (Additional file 1: Figure S1B).

### BMSCs attenuate ALF-related injury via induction of HO-1

**Fig. 1:**
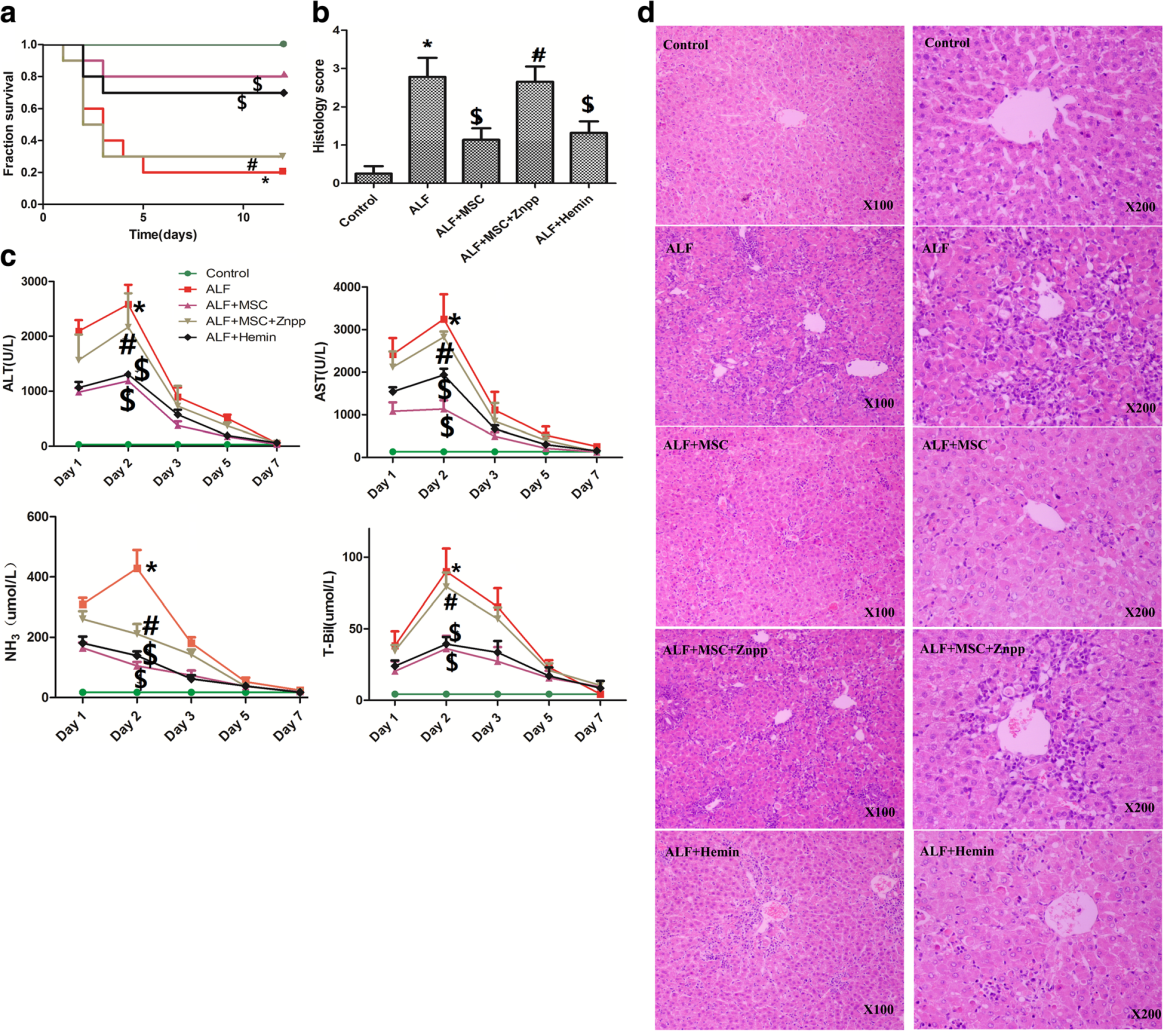
Transplanted BMSCs, via HO-1, significantly improve liver function, inflammation, and survival in ALF induced by D-Gal/LPS. **a** Kaplan-Meyer curve showing survival over 12 days (n = 10 per group) following various treatments. BMSCs significantly decreased mortality of ALF (*p* < 0.05), whereas Znpp treatment blocked this protective effect (*p* < 0.05). Hemin also resulted in a significant decrease in mortality (*p* < 0.05). **b** Liver injury scores (n = 6 per group) were significantly decreased by BMSCs (*p* < 0.05); Znpp reversed this protective effect (*p* < 0.05). Hemin treatment resulted in a decrease in injury score (*p* < 0.05). **c** Serum ALT, AST, NH_3_, and TBIL (n = 10 per group). BMSCs infusion significantly decreased liver enzymes over 7 days after infusion (*p* < 0.05); Znpp reversed this effect (*p* < 0.05). Hemin treatment resulted in a significant decrease in liver function tests (*p* < 0.05). **d** Representative images of hematoxylin and eosin staining (×100/×200). Treatment groups: control, ALF, ALF followed by intravenous MSCs (ALF + MSC) 1 h post-induction, ALF followed by MSCs and Znpp (ALF + MSC + Znpp) 1 h post-induction, and ALF followed by hemin (ALF + hemin) 1 h post-induction. Data are mean ± SD. (^*^ *p* < 0.05 vs. control group; ^$^ *p* < 0.05 vs. ALF group; ^#^ *p* < 0.05 vs. ALF + MSC group). *Abbreviations*: *ALF* acute liver failure, *ALT* alanine aminotransferase, *AST* aspartate transaminase, *BMSCs* bone marrow-derived mesenchymal stem cells, *D-Gal* d-Galactosamine, *HO-1* heme oxygenase-1, *LPS* lipopolysaccharide, *NH*_*3*_ ammonia, *TBIL* total bilirubin, *Znpp* zinc protoporphyrin

In the ALF group, 80% of rats died within 5 days. By contrast, 80% of rats receiving BMSCs survived for 12 days after ALF induction (Fig. 1a). All the changes were quantitatively evaluated using injury scores (Fig. 1b). The surviving animals also had a dramatic decrease in ALT, AST, total bilirubin (TBIL), and ammonia (NH_3_) levels (Fig. 1c). Histology showed a normal lobular architecture and cell structure of the liver in control animals, but extensive portal inflammation, hemorrhagic necrosis, increased inflammatory cell infiltration in the liver of the ALF group, and the hepatocytes had swollen cytoplasm. BMSCs treatment restored normal histology (Fig. 1d).

ALT, AST, TBIL, and NH_3_ were increased dramatically in the ALF group compared with the control group. To establish whether suppression of endogenous HO-1 activity affected the protective effect of BMSCs, we treated rats with ZnPP to suppress hepatic HO activity. We found that inhibition of HO-1 by ZnPP partly abolished the hepatoprotective effect of BMSCs, as confirmed by biochemical or histological parameters (Fig. 1c). The HO-1 induction by hemin in D-Gal/LPS-treated rats was investigated. D-Gal/LPS-induced hepatotoxicity was markedly attenuated in rats with hemin (Fig. 1).

**Fig. 2:**
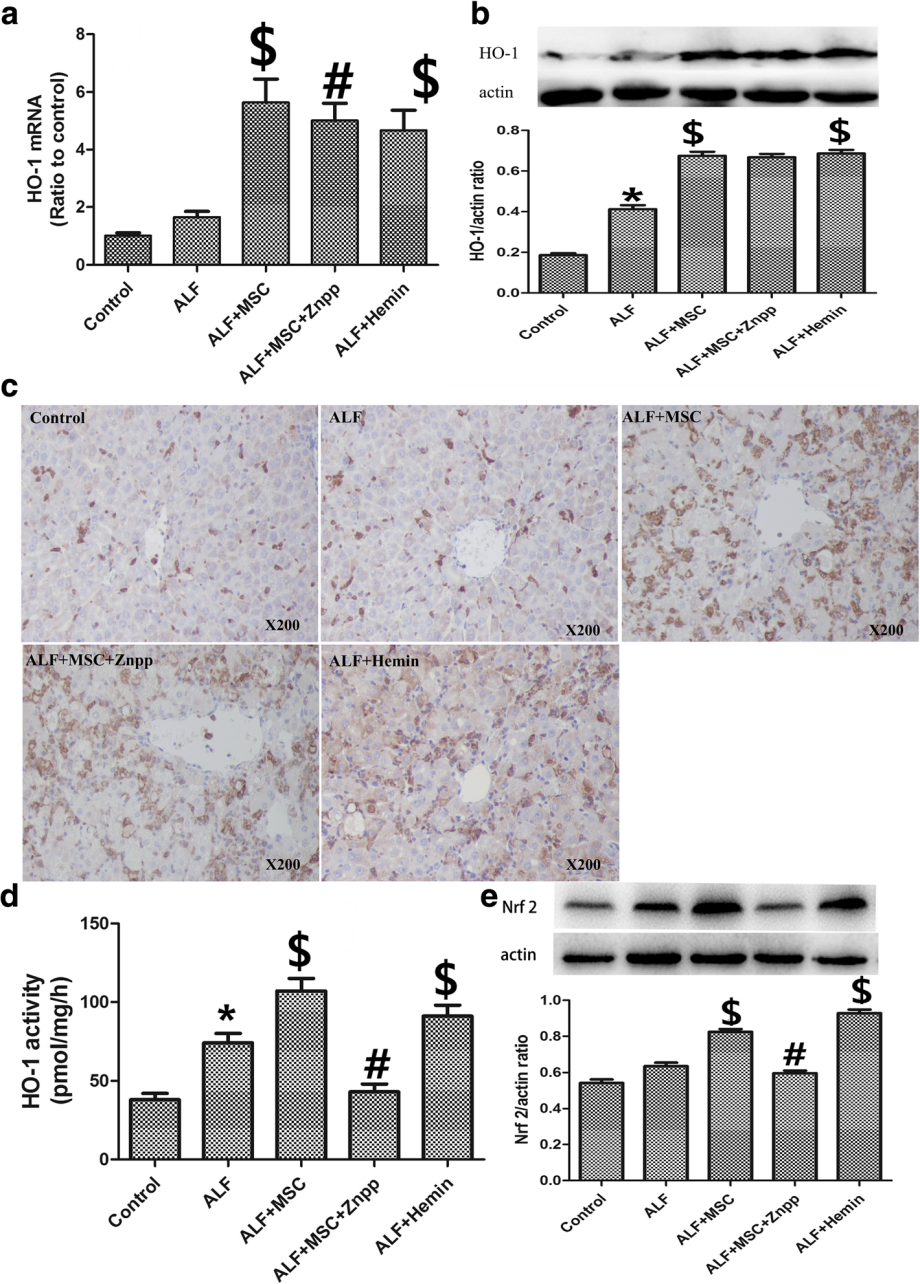
BMSCs increase HO-1 expression and activity, and Nrf2 expression in liver. **a** Quantitative reverse transcription PCR showing BMSC infusion or hemin increases expression of HO-1 relative to the ALF group (n = 6 per group, *p* < 0.05). Znpp did not decrease HO-1 mRNA levels. **b** Western blot shows that BMSCs infusion or hemin treatment significantly increases HO-1 protein (*p* < 0.05), but Znpp does not decrease HO-1 protein levels. Densitometry used to calculate normalized protein ratio (vs. β-actin standard). **c** Immunohistochemical stain (*brown*) of HO-1 in livers 72 h after D-Gal/LPS treatment (magnification × 200). BMSCs infusion or hemin treatment significantly increased HO-1 staining; Znpp did not decrease HO-1 staining. **d** HO-1 enzyme activity in livers 72 h after D-Gal/LPS treatment (n = 6 per group). HO-1 activity was significantly increased by BMSCs (*p* < 0.05); Znpp decreased HO-1 activity (*p* < 0.05). Hemin treatment also increased liver HO-1 activity (*p* < 0.05). **e** Western blot results showing that BMSCs increase Nrf2 expression of Nrf2 relative to the ALF group (*p* < 0.05); Znpp decreased Nrf2 expression (*p* < 0.05). Hemin treatment also increased Nrf2 protein levels (*p* < 0.05). Densitometry used to normalize protein levels (β-actin standard). Treatment groups: control, ALF, ALF followed by intravenous MSCs (ALF + MSC) 1 h post-induction, ALF followed by MSCs and Znpp (ALF + MSC + Znpp) 1 h post-induction, and ALF followed by hemin (ALF + hemin) 1 h post-induction. Data are mean ± SD. (^*^ *p* < 0.05 vs. control group; ^$^ *p* < 0.05 vs. ALF group; ^#^ *p* < 0.05 vs. ALF + MSC group). *Abbreviations*: *ALF* acute liver failure, *BMSCs* bone marrow-derived mesenchymal stem cells, *D-Gal* d-Galactosamine, *HO-1* heme oxygenase-1, *LPS* lipopolysaccharide, *Nrf2* nuclear factor-erythroid 2 p45-related factor 2, *Znpp* zinc protoporphyrin

Compared with the control and ALF groups, BMSCs and hemin induced a significant increase in HO-1 mRNA and protein expression in the liver (Fig. 2a–c). Immunohistochemical analysis showed the distribution of HO-1 protein after BMSC treatment. The positive staining of HO-1 protein was mainly observed in hepatic parenchymal cells (Fig. 2c). The control rats showed little positive staining in the liver, while the mRNA and protein levels of HO-1 were not affected by ZnPP. The HO-1 activity suggested an increased enzymatic function after BMSC and hemin treatment, and the increased HO-1 activity was reduced to baseline level by ZnPP (Fig. 2d). Nuclear factor-erythroid 2 p45-related factor 2 (Nrf2) is associated with cytoprotective genes, such as *HO*-*1* [20, 21]. As shown in Fig. 2e, MSCs transplantation significantly increased the expression of Nrf2.

### BMSCs inhibit neutrophil infiltration and oxidative activity via induction of HO-1

**Fig. 3:**
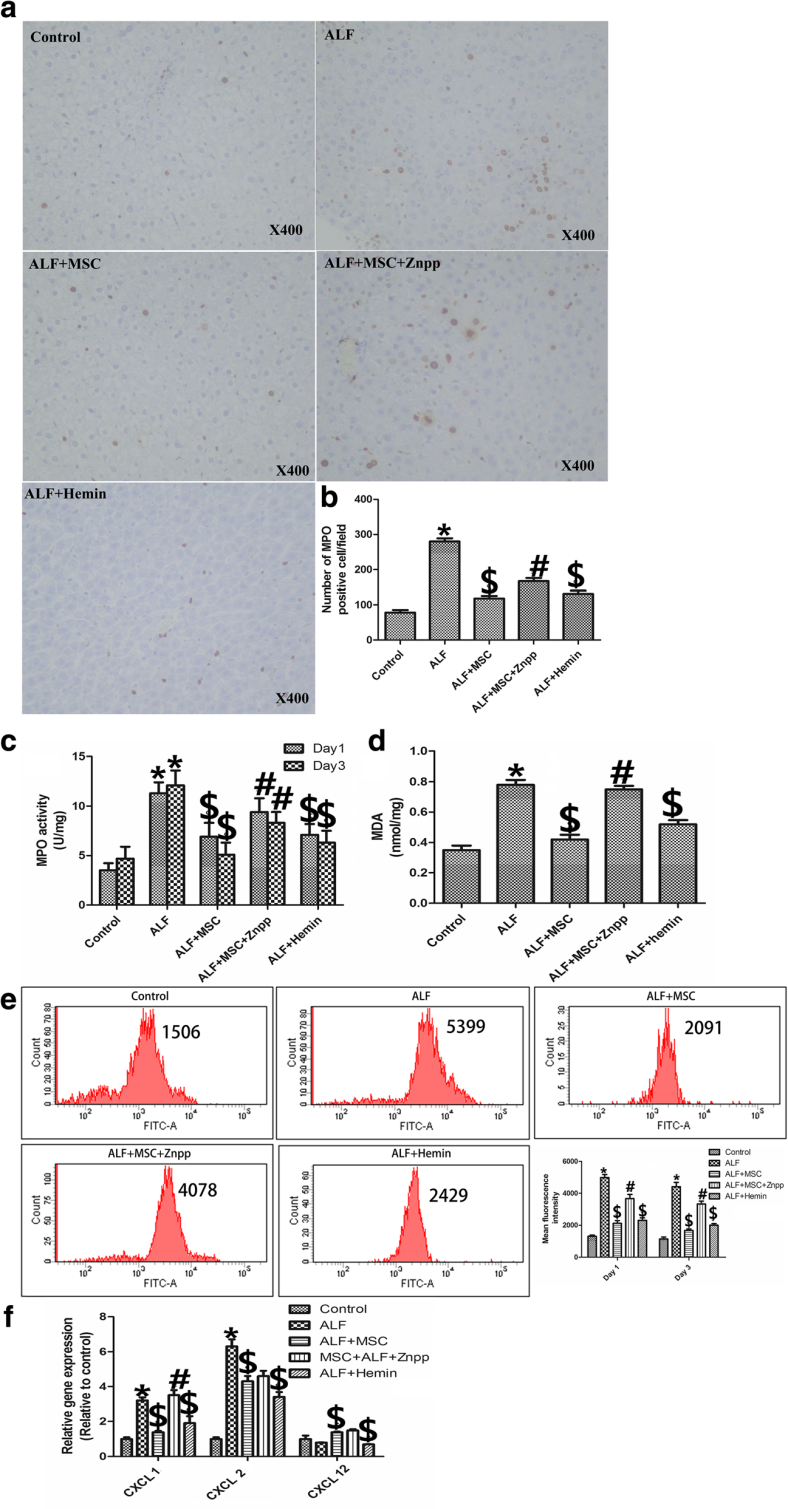
BMSCs inhibit neutrophil infiltration into liver, MPO activity, MDA expression, and neutrophil oxidative burst via HO-1 induction in D-Gal/LPS-induced ALF. **a** Myeloperoxidase (MPO, a neutrophil marker) staining (*brown*) in livers 72 h after D-Gal/LPS (magnification × 400). **b** Number of MPO-positive cells in each group (n = 6 per group). ALF rats had the most MPO-positive cells, BMSCs infusion significantly decreased the number of MPO-positive cells (*p* < 0.05). Znpp resulted in more MPO- + cells than BMSC alone (*p* < 0.05). **c** MPO activity was significantly increased by D-Gal/LPS vs. control rats (*p* < 0.05). BMSCs decreased MPO activity induced by ALF (*p* < 0.05); Znpp blunted the BMSC effect. Hemin treatment after BMSC was also associated with reduced MPO activity (*p* < 0.05) (n = 6 per group). Livers collected 24 and 72 h following D-Gal/LPS. **d** Malondialdehyde (MDA) levels (n = 6 per group) were increased significantly in ALF livers. BMSCs significantly reduced MDA expression after ALF (*p* < 0.05). Znpp administration blunted the effect of BMSC (*p* < 0.05). Hemin treatment after BMSC was also associated with decreased MDA levels (*p* < 0.05). **e** R123 fluorescence of neutrophils (oxidative stress) at 24 h and 72 h after D-Gal/LPS (n = 6 per group). Fluorescence intensity increased significantly in ALF livers. BMSCs infusion resulted in a significant reduction of fluorescence intensity (*p* < 0.05), whereas Znpp administration blunted the BMSC effect on fluorescence (*p* < 0.05). Hemin after BMSC was also associated with decreased fluorescence vs. ALF (*p* < 0.05), **f** Quantitative reverse transcription PCR for mRNA levels of CXCL1, CXCL2, and CXCL12 in livers. CXCL1 and CXCL2 expression increased significantly in ALF livers (*p* < 0.05). BMSCs infusion decreased the mRNA levels of CXCL1 and CXCL2 vs. ALF levels (*p* < 0.05); Znpp administration blunted the effects of BMSCs only on CXCL1 mRNA expression (*p* < 0.05). Hemin treatment after BMSC was also associated with decreased CXCL1 and CXCL2 mRNA vs. ALF (*p* < 0.05). Treatment groups: control, ALF, ALF followed by intravenous MSCs (ALF + MSC) 1 h post-induction, ALF followed by MSCs and Znpp (ALF + MSC + Znpp) 1 h post-induction, and ALF followed by hemin (ALF + hemin) 1 h post-induction. Data are mean ± SD. (^*^ *p* < 0.05 vs. control group; ^$^ *p* < 0.05 vs. ALF group; ^#^ *p* < 0.05 vs. ALF + MSC group). *Abbreviations*: *ALF* acute liver failure, *BMSCs* bone marrow-derived mesenchymal stem cells, *D-Gal* d-Galactosamine, *FITC* fluorescein isothiocyanate, *HO-1* heme oxygenase-1, *LPS* lipopolysaccharide, *Znpp* zinc protoporphyrin

Neutrophils play an important role in ALF, and pro-inflammatory cytokines promote their activation and infiltration in the liver, during chemokine and adhesion molecule expression [22, 23]. Therefore, we examined the effect of BMSCs on neutrophil infiltration. Cells positive for MPO (a neutrophil marker) were observed in liver. The number of MPO-positive cells, MPO activity and MDA level were markedly increased in the ALF group and were downregulated by BMSC treatment. The inhibitory effect of BMSCs in neutrophils was attenuated by addition of ZnPP, and hemin also inhibited neutrophil infiltration and activity (Fig. 3).

To assess the oxidative burst, neutrophils were gated, using flow cytometry, by their characteristic forward and side scatter profiles and analyzed for fluorescence intensity (Additional files 2 and 3: Figure S4 and Figure S5). Blood sample gating is illustrated in Fig. 3. Fluorescence-activated cell sorting results were presented as histograms representing the number of cells. The intensity of R123 fluorescence generated by incubating blood samples with DHR123 is shown in Fig. 3e. At 1, 2, and 3 days after injury, D-Gal/LPS-treated rats showed increased intensity of R123 fluorescence in neutrophils due to oxidative burst, which was notably alleviated by BMSCs or hemin treatment. The inhibitory effect of BMSCs on R123 fluorescence intensity of neutrophils was attenuated after addition of ZnPP. We also determined the impact of MSCs on the expression of chemokines CXCL1, CXCL2, and CXCL12 in the liver using RT-PCR. D-Gal/LPS increased the expression of *CXCL1* and *CXCL2* genes significantly, which induced PMN infiltration into the liver, while the *CXCL12* expression declined slightly. Transplantation of MSCs led to a significant decrease in CXCL1 and CXCL2 expression, similar to the events following HO-1 induction. However, HO-1 inhibition increased CXCL1, but not CXCL2 expression (Fig. 3f).

### BMSCs reduced ALF-related apoptosis and promoted regeneration via induction of HO-1

**Fig. 4:**
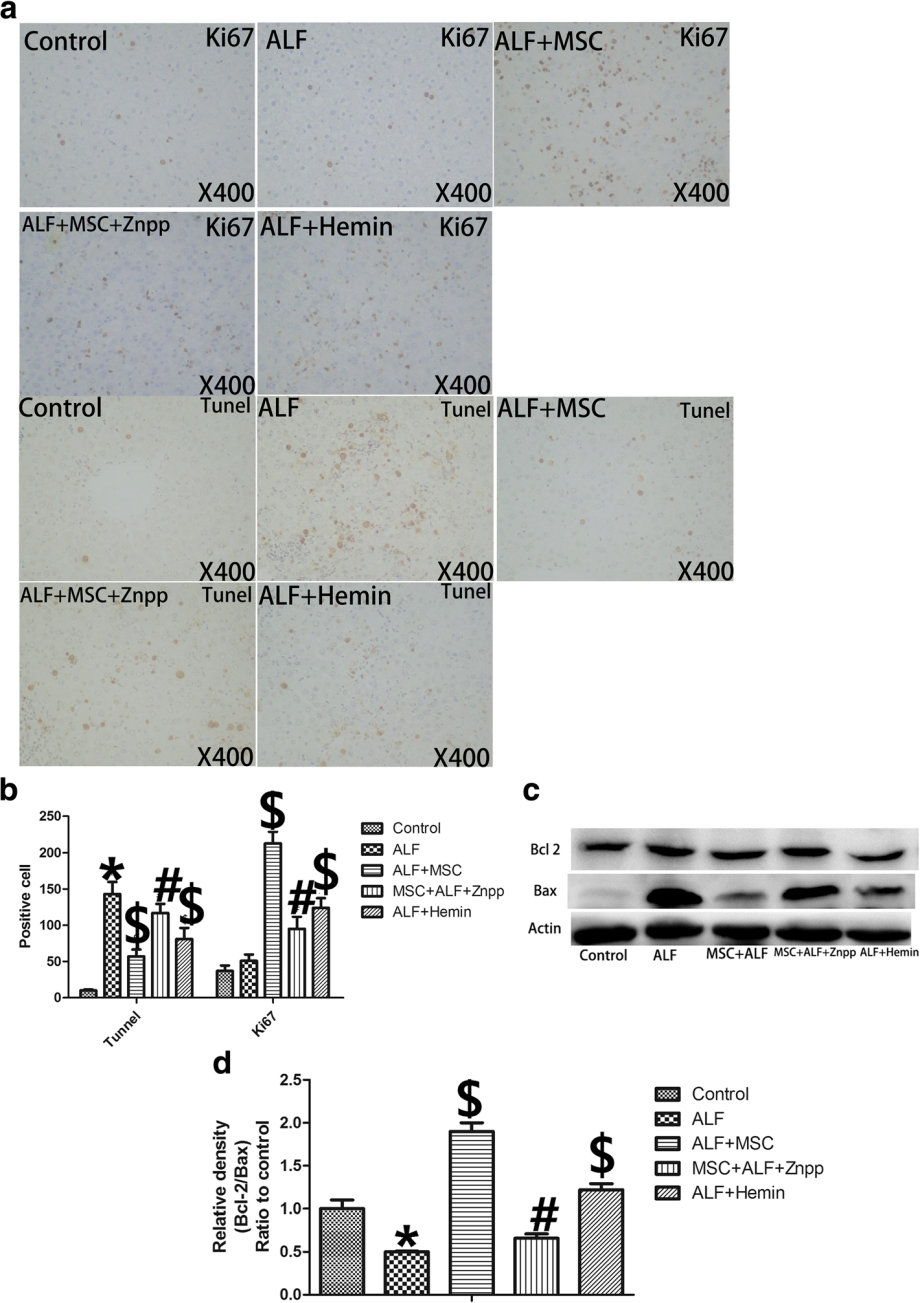
BMSCs reduce hepatocyte apoptosis and promote proliferation via induction of HO-1 in the setting of ALF. **a** TUNEL (marking apoptosis) and Ki67 (a proliferation marker) in livers 72 h after D-Gal/LPS (magnification × 200). **b** Numbers of TUNEL- + and Ki67- + cells in each group (*n* = 6 per group). ALF livers had the highest number of TUNEL- + cells. BMSCs significantly reduced numbers of TUNEL- + cells (*p* < 0.05); Znpp blunted the anti-apoptotic effect of BMSCs (*p* < 0.05). BMSC and hemin also resulted in significantly less TUNEL- + cells vs. ALF (*p* < 0.05). BMSCs significantly increased the number of ki67- + cells relative to ALF livers (*p* < 0.05); Znpp blunted the BMSC effect on proliferation (*p* < 0.05). BMSC plus hemin also increased the number of ki67-positive cells vs. ALF livers (*p* < 0.05). **c** Western blot of Bcl-2 and Bax protein levels (normalized to β-actin standard). **d** The Bcl-2/Bax ratio decreased significantly in ALF livers. BMSCs infusion increased the Bcl-2/Bax ratio (*p* < 0.05); Znpp blunted this effect of BMSCs (*p* < 0.05). BMSCs plus hemin treatment also increased Bcl-2/Bax ratios vs. ALF livers (*p* < 0.05). Treatment groups: control, ALF, ALF followed by intravenous MSCs (ALF + MSC) 1 h post-induction, ALF followed by MSCs and Znpp (ALF + MSC + Znpp) 1 h post-induction, and ALF followed by hemin (ALF + hemin) 1 h post-induction. Data are mean ± SD. (^*^ *p* < 0.05 vs. control group; ^$^ *p* < 0.05 vs. ALF group; ^#^ *p* < 0.05 vs. ALF + MSC group). *Abbreviations*: *ALF* acute liver failure, *BMSCs* bone marrow-derived mesenchymal stem cells, *D-Gal* d-Galactosamine *HO-1* heme oxygenase-1, *LPS* lipopolysaccharide, *TUNEL* 2’-deoxyuridine 5’-triphosphatenick-end labeling, *Znpp* zinc protoporphyrin

Recent studies demonstrated that BMSCs decreased apoptosis in damaged liver. To determine the role of HO-1 in this process, we investigated the effect of BMSCs on ALF-related apoptosis. We found a significant increase in the number of TUNEL-positive cells (Fig. 4a) in the ALF group compared with the control group, which was reduced markedly 2 days after BMSCs or hemin treatment. The balance between the expression of anti-apoptotic protein Bcl-2 and pro-apoptotic protein Bax not only regulates apoptosis but also served as an important indicator of apoptosis [25]. Western blot (Fig. 4c) showed that the Bcl-2/Bax ratio was reduced in ALF compared with the control rats, and was upregulated 2 days after BMSC or hemin treatment. The Bcl-2/Bax ratio was downregulated by ZnPP treatment.

To determine the effect of BMSCs on liver regeneration, the number of Ki-67-positive hepatocytes were counted and compared. Ki-67-positive cells were considered to be proliferative. BMSCs significantly upregulated the number of Ki-67-positive hepatocytes compared with the control and ALF groups (Fig. 4a), which was decreased by the addition of ZnPP. Hemin also promoted the number of Ki-67-positive hepatocytes, but the number of Ki-67-positive hepatocytes was lower than that of BMSCs (Fig. 4b).

### BMSCs decreased inflammatory response via induction of HO-1

**Fig. 5:**
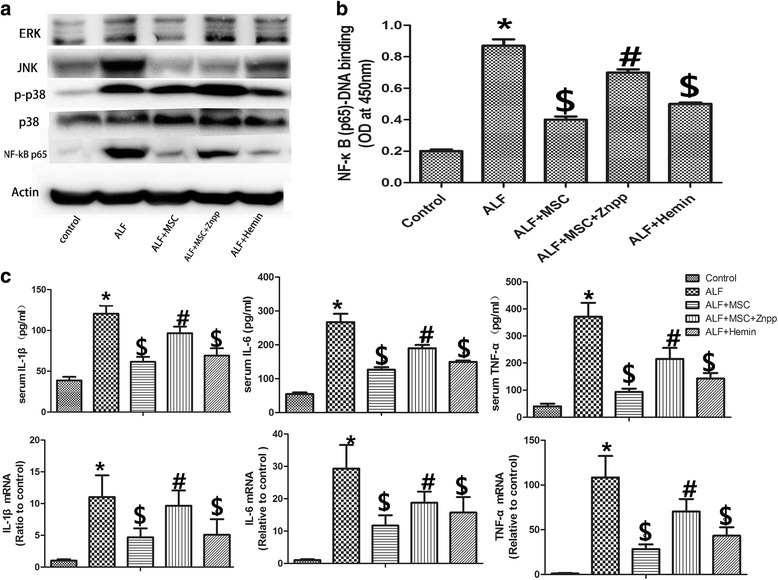
BMSCs decrease inflammatory cytokines via HO-1 in the setting of ALF. **a** Western blot of phosphorylated P38 mitogen-activated protein kinase (MAPK), extracellular-regulated protein kinases (Erk), C-Jun N-terminal kinase (JNK), nuclear factor kappa B (NF-κB) p65 (and β-actin standard). Levels of JNK, p-p38, NF-κB p65 increased in ALF livers vs. controls. BMSCs decreased ALF levels of JNK, NF-κB p65; Znpp only blunted the BMSC inhibition of NF-κB p65 expression. Hemin treatment also resulted only in reduction of NF-kB p65 protein. **b** NF-κB DNA binding activity (*n* = 6 per group) increased significantly in ALF livers. BMSCs significantly reduced NF-κB DNA binding activity (*p* < 0.05). Znpp administration could reverse this BMSC effect (*p* < 0.05). Hemin and BMSC were also associated with decreased NF-κB DNA binding activity vs. ALF livers (*p* < 0.05). **c** Enzyme-linked immunosorbent assay of serum levels of IL-1β, IL-6, and TNF-α after D-Gal/LPS (n = 6 per group). Quantitative reverse transcription PCR for IL-1β, IL-6, and TNF-α mRNA levels in livers. BMSCs infusion significantly decreased protein or mRNA levels of IL-1β, IL-6, and TNF-α (*p* < 0.05); Znpp administration reversed this effect of the BMSCs (*p* < 0.05). Hemin and BMSCs were also associated with decreased protein and mRNA levels of IL-1β, IL-6, and TNF-α (*p* < 0.05) vs. ALF livers. Treatment groups: control, ALF, ALF followed by intravenous MSCs (ALF + MSC) 1 h post-induction, ALF followed by MSCs and Znpp (ALF + MSC + Znpp) 1 h post-induction, and ALF followed by hemin (ALF + hemin) 1 h post-induction. Data are mean ± SD. (^*^ *p* < 0.05 vs. control group; ^$^ *p* < 0.05 vs. ALF group; ^#^ *p* < 0.05 vs. ALF + MSC group). *Abbreviations*: *ALF* acute liver failure, *BMSCs* bone marrow-derived mesenchymal stem cells, *D-Gal* d-Galactosamine, *HO-1* heme oxygenase-1, *IL* interleukin, *LPS* lipopolysaccharide, *TNF* tumor necrosis factor, *Znpp* zinc protoporphyrin

Pro-inflammatory cytokines play an important role in liver injury [24]. Phosphorylation of the NF-κB p65 subunit is associated with an increase in NF-κB activity, and plays an important inflammatory role [25]. As shown in Fig. 5a, D-Gal/LPS injection significantly induced phosphorylation of NF-κB p65 subunit. BMSCs markedly reduced the phosphorylation of NF-κB p65 subunit. Following the inhibition of HO-1 activity in liver, the phosphorylation of NF-κB p65 subunit increased again. Hemin decreased the phosphorylation of NF-κB p65 subunit. To investigate whether BMSCs affected NF-κB DNA-binding activity, an NF-κB activity assay was performed. The D-Gal/LPS-induced increase in DNA binding of NF-κB, BMSCs and hemin downregulated the DNA binding of NF-κB, and ZnPP increased the NF-κB DNA binding activity (Fig. 5b).

Mitogen-activated protein kinases (MAPKs) play an important role in the control of cellular responses to inflammatory stimuli. After induction of ALF, D-Gal/LPS resulted in the upregulation of phosphorylation of p38, extracellular-regulated protein kinases (Erk) and C-Jun N-terminal kinase (JNK), and BMSC transplantation attenuated JNK expression. Increased phosphorylation of p38 was minimally affected by BMSCs, and inhibition of HO-1 activity did not affect the phosphorylation of JNK, while hemin increased the phosphorylation of p38 and JNK. The levels of IL-6 in liver (Fig. 5c), as well as IL-1B and TNF-α were elevated in the ALF group compared with the control group. The expression of pro-inflammatory cytokines was decreased after BMSCs or hemin treatment, and inhibition of HO-1 activity with ZnPP increased the levels of IL-6, IL-1β, and TNF-α compared with BMSC treatment. Increased mRNA levels of IL-1β, IL-6, and TNF-α (Fig. 5c) were also detected, especially in the ALF group. BMSCs or hemin treatment downregulated the mRNA level of IL-1β, IL-6, and TNF-α. However, the anti-inflammatory effect of BMSC was partly abrogated by ZnPP treatment.

## Discussion

MSCs have been studied for the treatment of liver failure for several years. The possible mechanisms are mediated via complex paracrine pathways, underscoring their significance as an important cellular source in liver regenerative medicine. Previous studies suggested that rodent or human MSCs differentiate into hepatocytes in vitro*.* However, the role of MSCs transplantation in regeneration of hepatocytes remains controversial [26]. The majority of recent studies indicated that the trophic and immunomodulatory factors secreted by MSCs play a crucial role in liver injury [4, 5, 12, 27]. In the current study, we demonstrated that MSCs increased the mRNA and protein levels of HO-1 in liver, which led to significant improvement in liver injury, inflammatory response, and neutrophil infiltration. In vivo imaging and fluorescence microscopy demonstrated that Dir-labeled MSCs were detected in the liver (Additional files 4 and 5: Figure S2 and Figure S3). These findings show that MSCs home to sites of liver injury. Taken altogether, our study suggests that MSCs treatment alleviates ALF through the induction of HO-1.

HO-1 is a stress-response protein, which is upregulated by a broad spectrum of inducers, including heme, heavy metals, nephrotoxins, cytokines, endotoxins, and oxidative stress [13, 28, 29]. Nrf2 is a transcription factor mediating the Nrf2-antioxidant response element signaling pathway, which protects against oxidative stress. Therefore, Nrf2 plays an important therapeutic role in inflammatory diseases [20, 21, 30]. Our results suggested that MSC transplantation might exert a protective effect by activating the Nrf2 pathway. The present study showed that the hepatic expression of Nrf2 and its target gene, *HO*-*1*, were significantly increased following treatment with BMSCs. HO-1 activity was also markedly induced by BMSCs. The data confirm the HO-1-inducing property of BMSCs [13, 31]. However, the mechanism involved in the activation of HO-1 protein expression by BMSCs in rats remains to be investigated.

Recent studies have demonstrated that HO-1 regulated neutrophil infiltration and activation. Konrad​ et al. [32] showed that the anti-inflammatory role of HO-1 is mediated via inhibition of neutrophil infiltration from bone marrow. Konrad et al. [33] attributed the anti-inflammatory effects of HO-1 primarily to inhibition of PMN release from bone marrow. Neutrophils are an important group of innate immune cells in the first line of defense against disease. Neutrophils are generated in bone marrow and released into the circulation. They are rapidly recruited to the sites of injury and inflammation [34]. During liver injury in ALF, neutrophils are activated by pro-inflammatory cytokines and increase tissue damage by enhanced oxidative burst and reactive oxygen species [35]. Excessive neutrophil infiltration and activation can have adverse effects. Consistent with previous studies [35], our findings suggest that injection of D-Gal/LPS increase the migration of neutrophils, elevate their oxidative burst levels, enhance hepatic MPO activity, and upregulate MDA expression. These effects were downregulated by BMSCs. The protective effect of BMSCs was attenuated after addition of ZnPP. The excessive activation and infiltration of neutrophils leads to secondary inflammation in the liver or bystander tissues [36, 37]. Our findings are consistent with other studies reporting that MSCs dampen oxidative and inflammatory activity of neutrophils of premature neonates in vitro [38]. The chemokines CXCL1 (keratinocyte-derived chemokine) and CXCL2 (macrophage inflammatory protein-2) are crucial for neutrophil migration to inflammatory sites [32]. CXCL1 and CXCL2 are 90% identical in their amino acid sequences and act via the chemokine receptor CXCR2, which is a G protein chemokine receptor expressed on neutrophils [39]. Hepatic CXCL1 mRNA levels correlated positively with liver PMN infiltration in the alcoholic hepatitis period [40]. Our results showed that MSCs transplantation significantly suppressed *CXCL1* and *CXCL2* genes, while inhibition of the HO-1 restored the CXCL1 levels, suggesting that CXCL1 mediated the protective effect of HO-1 after MSCs transplantation.

ALF is associated with significant inflammation. We evaluated the effects of BMSCs on cytokines such as IL-1β, IL-6, and TNF-α, which are biomarkers closely associated with severe inflammation [6]. Our study showed that BMSCs significantly inhibited the expression of IL-1β, IL-6, and TNF-α induced by D-Gal/LPS, and HO-1 played a key anti-inflammatory role. Downregulation of HO-1 is associated with exacerbation of inflammatory response by TNF-α in human monocytes [25]. In chronic ethanol-induced liver injury, Bakhautdin et al. [41] reported attenuation of TNF-α-induced cell death and oxidative stress via HO-1. Several pathological factors trigger NF-κB signaling, which regulates the expression of inflammatory cytokines. Our study showed that BMSCs markedly inhibited the activation of NF-κB via induction of HO-1. MAPKs play an important role in the regulation of D-Gal-induced inflammation by controlling NF-κB activation [42]. We demonstrated that BMSCs inhibited JNK in response to D-Gal/LPS, suggesting that MAPKs mediate the suppression of BMSC-induced NF-κB activation by D-Gal/LPS, which also explains the inhibition of neutrophils during the anti-inflammatory response.

Our study limitations are as follows. First, the potential participation of factors/pathways between MSCs and HO-1 has not been clarified, although our research suggest an important role for Nrf2. The identifying task would be time-consuming because it requires knockout or other similar experiments. Next, our study was preliminary because it was not carried out for a longer period of time, as the ideal of MSCs alone as a liver dialysis would not be expected to extend survival. In spite of these limitations, our work provides a novel message between BMSCs and HO-1 in ALF rats.

## Conclusions

We demonstrated that BMSCs ameliorated ALF by increasing the expression of HO-1, and reducing PMN infiltration and function. BMSCs play an important anti-inflammatory and anti-apoptotic role.

## Additional files

Additional file 1: Figure S1. Characterization of BMSCs. (A) Morphology of BMSCs at day 12 of culture. Isolated BMSCs grow as fibroblast-like cells. Magnification × 50 and × 100. Scale bars = 100 μm. (B) MSC marker profile of BMSCs. immunophenotype of BMSCs is determined by flow cytometry with the use of labeled antibodies specific for the known markers (CD29, CD34, CD44, CD45, and CD90), BMSCs isolated from rat were positive for CD29 (98.9%), CD44 (97.1%), and CD90 (99.2%) and negative for CD34 (0.8%) and CD45 (2.0%), which meant high purity after the third passage. Abbreviations: *BMSCs* bone marrow mesenchymal stem cells, *CD* cluster of differentiation, *PE* P-phycoerythrin, *FITC* fluorescein isothiocyanate. (JPG 7121 kb)
Additional file 2: Figure S4. Gating strategy for neutrophils. Example of gating of a blood sample for flow cytometry using physical characteristics of granularity (FSC) and size (SSC). Neutrophils constituted the largest population. (JPG 149 kb)
Additional file 3: Figure S5. Gating strategy for neutrophils. Neutrophils were further gated to determine purity (CD11b FITC and His 48 PE). (JPG 92 kb)
Additional file 4: Figure S2. In vivo fluorescence imaging of rats 3 days after injection with Dir-labeled mesenchymal stem cells. Strong fluorescence was detected in the liver showing that BMSCs home to the injured liver. Treatment groups: control, ALF, ALF followed by intravenous MSCs (ALF + MSC) 1 h post-induction, ALF followed by MSCs and Znpp (ALF + MSC + Znpp) 1 h post-induction, and ALF followed by hemin (ALF + hemin) 1 h post-induction. Data are mean ± SD. (^*^ *p* < 0.05 vs. control group; ^$^ *p* < 0.05 vs. ALF group; ^#^ *p* < 0.05 vs. ALF + MSC group). (JPG 2007 kb)
Additional file 5: Figure S3. Fluorescence microscopy of Dir-labeled BMSCs engrafted in liver tissues, detected by fluorescence microscope. (A) Dir-labeled BMSCs were detected in liver lobular parenchyma 3 days post-transplantation. (B) Quantification of BMSCs in the livers of ALF + MSC and ALF + MSC + Znpp groups. The distribution of Dir-labeled BMSCs in liver tissues was observed by fluorescence microscope. Fluorescence microscope exhibited that in ALF + MSC group and ALF + MSC + Znpp group, MSC distributed in liver dispersedly. (JPG 4233 kb)

## Abbreviations

ALF: Acute liver failure
ALT: Alanine aminotransferase
AST: Aspartate transaminase
BMSCs: Bone marrow-derived mesenchymal stem cells
CD: Cluster of differentiation
D-Gal: d-Galactosamine
Erk: Extracellular-regulated protein kinase
FITC: Fluorescein isothiocyanate
HO: Heme oxygenase
IL: Interleukin
JNK: C-Jun N-terminal kinase
LPS: Lipopolysaccharide
MAPKs: Mitogen-activated protein kinases
MDA: Malondialdehyde
MPO: Myeloperoxidase
MSCs: Mesenchymal stem cells
NF: nuclear factor
Nrf2: Nuclear factor-erythroid 2 p45-related factor 2
OLT: Orthotopic liver transplantation
PE: P-phycoerythrin
PMN: Polymorphonuclear neutrophil
TBIL: Total bilirubin
TNF: Tumor necrosis factor
TUNEL: 2’-deoxyuridine 5’-triphosphatenick-end labeling
ZnPP: Zinc protoporphyrin
