# Supplementary figures and images for: Mesenchymal stem cells increase expression of heme oxygenase-1 leading to anti-inflammatory activity in treatment of acute liver failure
Source: Stem Cell Res Ther. 2017 Mar 20;8:70. doi: 10.1186/s13287-017-0524-3 (PMC5359839; doi:10.1186/s13287-017-0524-3)

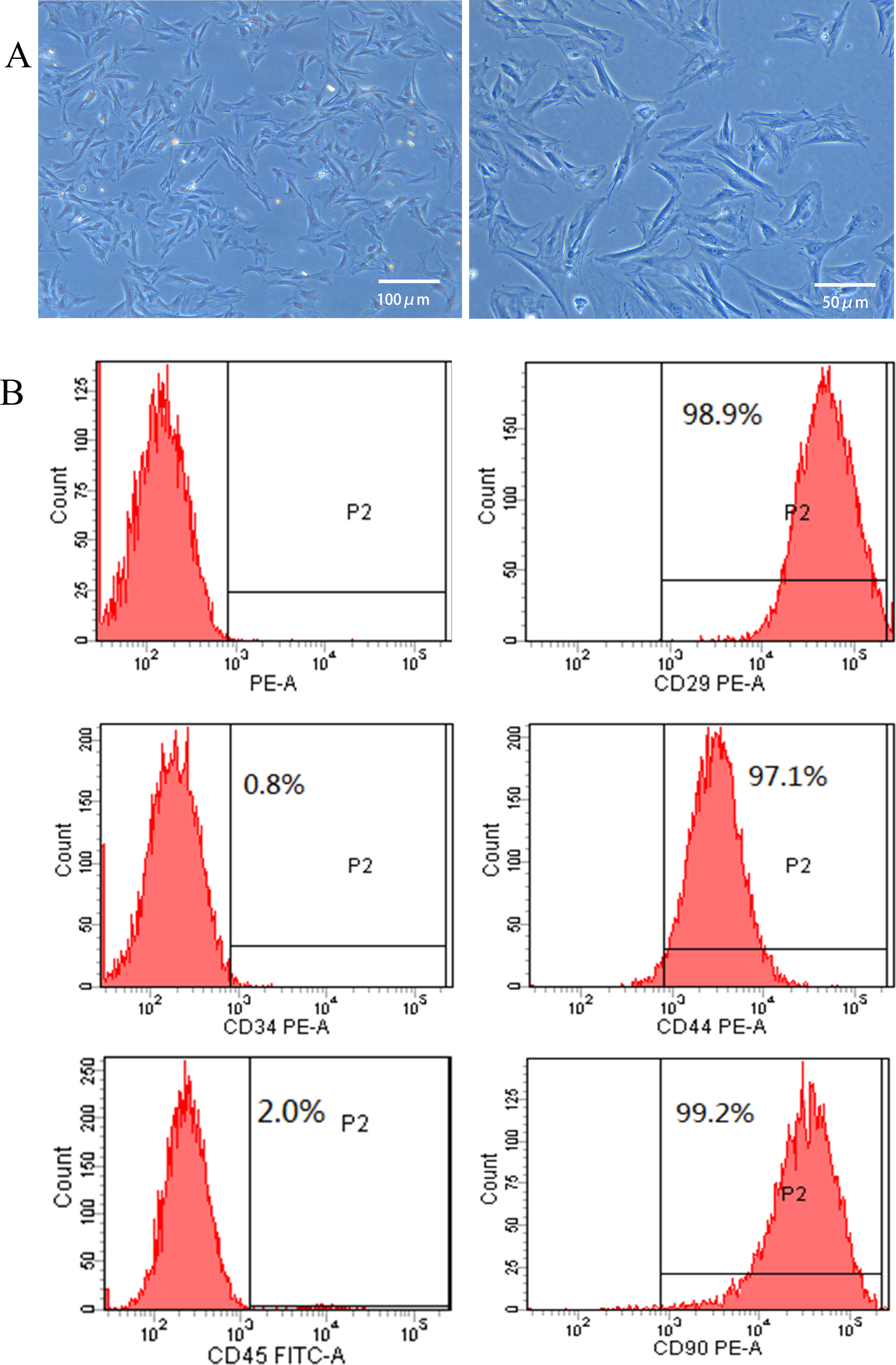

Supplement: Additional file 1: Figure S1. — Characterization of BMSCs. (A) Morphology of BMSCs at day 12 of culture. Isolated BMSCs grow as fibroblast-like cells. Magnification × 50 and × 100. Scale bars = 100 μm. (B) MSC marker profile of BMSCs. immunophenotype of BMSCs is determined by flow cytometry with the use of labeled antibodies specific for the known markers (CD29, CD34, CD44, CD45, and CD90), BMSCs isolated from rat were positive for CD29 (98.9%), CD44 (97.1%), and CD90 (99.2%) and negative for CD34 (0.8%) and CD45 (2.0%), which meant high purity after the third passage. Abbreviations: BMSCs bone marrow mesenchymal stem cells, CD cluster of differentiation, PE P-phycoerythrin, FITC fluorescein isothiocyanate. (JPG 7121 kb) [file 13287_2017_524_MOESM1_ESM.jpg]

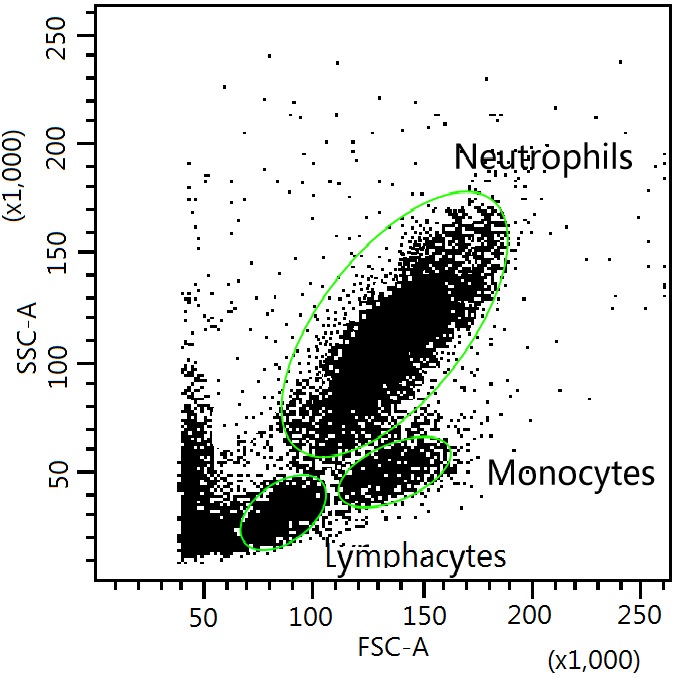

Supplement: Additional file 2: Figure S4. — Gating strategy for neutrophils. Example of gating of a blood sample for flow cytometry using physical characteristics of granularity (FSC) and size (SSC). Neutrophils constituted the largest population. (JPG 149 kb) [file 13287_2017_524_MOESM2_ESM.jpg]

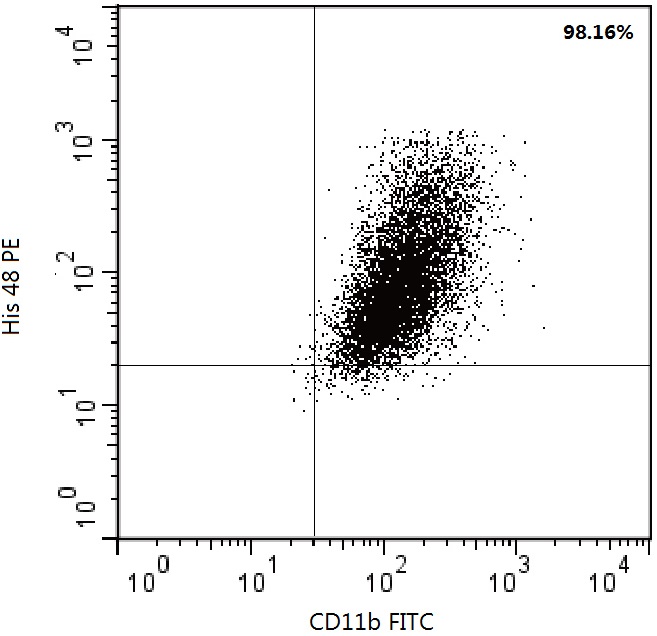

Supplement: Additional file 3: Figure S5. — Gating strategy for neutrophils. Neutrophils were further gated to determine purity (CD11b FITC and His 48 PE). (JPG 92 kb) [file 13287_2017_524_MOESM3_ESM.jpg]

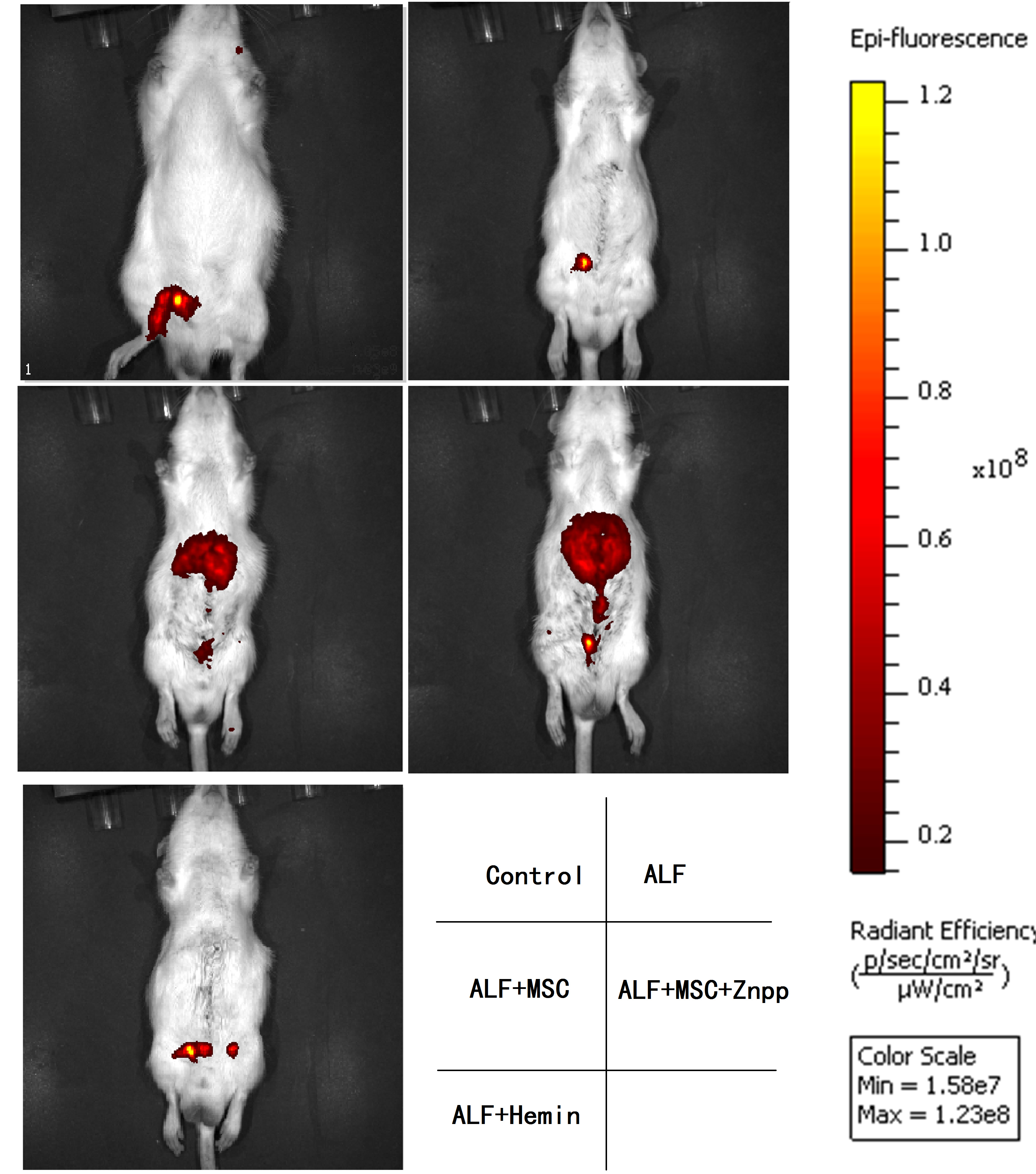

Supplement: Additional file 4: Figure S2. — In vivo fluorescence imaging of rats 3 days after injection with Dir-labeled mesenchymal stem cells. Strong fluorescence was detected in the liver showing that BMSCs home to the injured liver. Treatment groups: control, ALF, ALF followed by intravenous MSCs (ALF + MSC) 1 h post-induction, ALF followed by MSCs and Znpp (ALF + MSC + Znpp) 1 h post-induction, and ALF followed by hemin (ALF + hemin) 1 h post-induction. Data are mean ± SD. (* p < 0.05 vs. control group; $ p < 0.05 vs. ALF group; # p < 0.05 vs. ALF + MSC group). (JPG 2007 kb) [file 13287_2017_524_MOESM4_ESM.jpg]

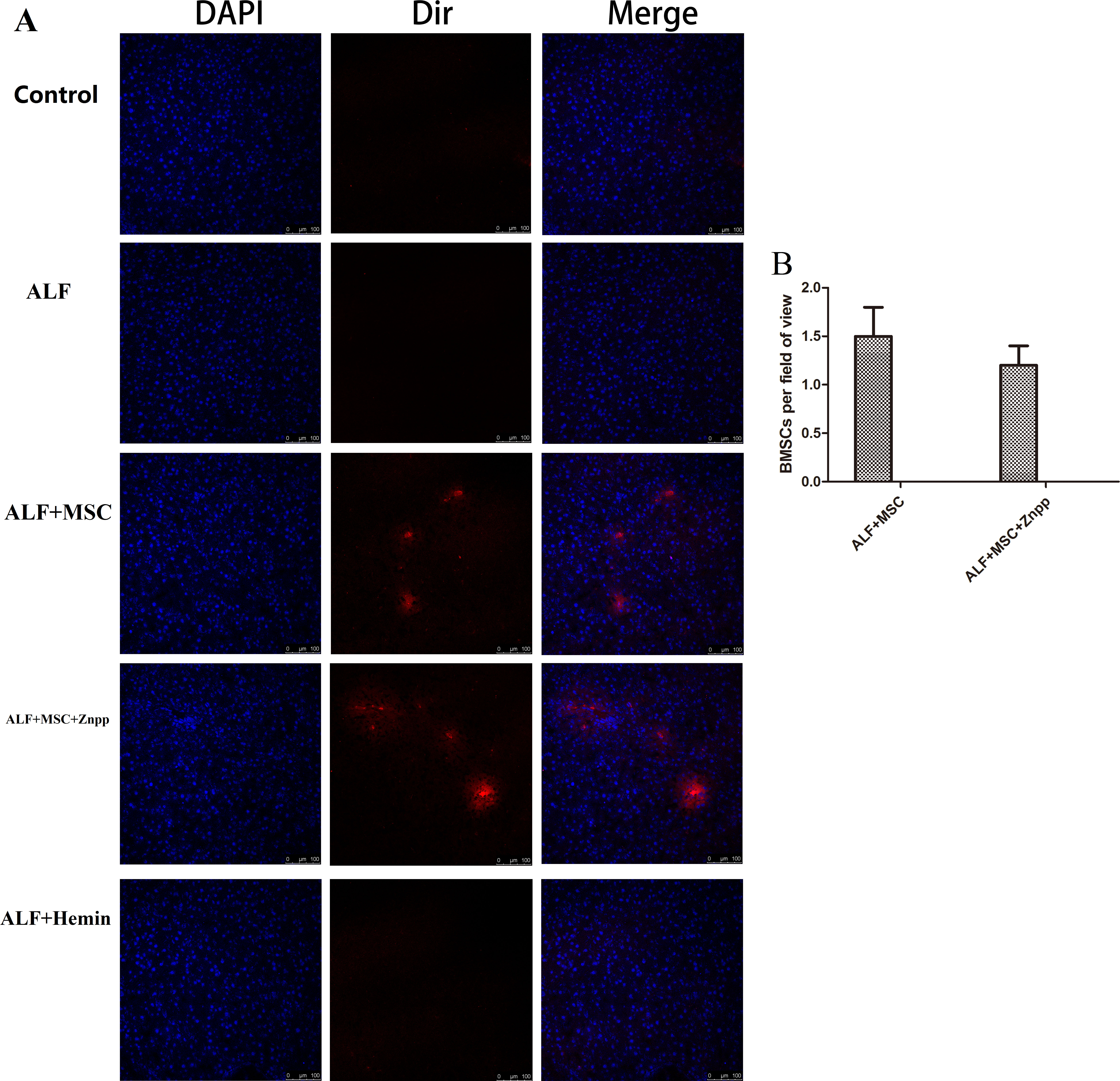

Supplement: Additional file 5: Figure S3. — Fluorescence microscopy of Dir-labeled BMSCs engrafted in liver tissues, detected by fluorescence microscope. (A) Dir-labeled BMSCs were detected in liver lobular parenchyma 3 days post-transplantation. (B) Quantification of BMSCs in the livers of ALF + MSC and ALF + MSC + Znpp groups. The distribution of Dir-labeled BMSCs in liver tissues was observed by fluorescence microscope. Fluorescence microscope exhibited that in ALF + MSC group and ALF + MSC + Znpp group, MSC distributed in liver dispersedly. (JPG 4233 kb) [file 13287_2017_524_MOESM5_ESM.jpg]
